# The roles of the tumor suppressor parafibromin in cancer

**DOI:** 10.3389/fcell.2022.1006400

**Published:** 2022-09-21

**Authors:** Hua-chuan Zheng, Hang Xue, Cong-yu Zhang

**Affiliations:** ^1^ Department of Oncology and Central Laboratory, The Affiliated Hospital of Chengde Medical University, Chengde, China; ^2^ Cancer Center, The First Affiliated Hospital of Jinzhou Medical University, Jinzhou, China

**Keywords:** parafibromin, cancer, tumor suppressor, tumorigenesis, hyperparathyroidism-jaw tumor (HPT-JT) syndrome

## Abstract

In this review, we discuss parafibromin protein, which is encoded by *CDC73*. A mutation in this gene causes hyperparathyroidism-jaw tumor (HPT-JT) syndrome, an autosomal dominant disease. *CDC73* is transcriptionally downregulated by the Wilms’ tumor suppressor gene WT1 and translationally targeted by miR-182-3p and miR-155. In the nucleus, parafibromin binds to RNA polymerase II and PAF1 complex for transcription. Parafibromin transcriptionally increases the expression of c-Myc, decreases CPEB1 expression by interacting with H3M4, and reduces cyclin D1 expression by binding to H3K9. The RNF20/RNF40/parafibromin complex induces monoubiquitination of H2B-K120, and SHP2-mediated dephosphorylation of parafibromin promotes the parafibromin/β-catenin interaction and induces the expression of Wnt target genes, which is blocked by PTK6-medidated phosphorylation. Parafibromin physically associates with the CPSF and CstF complexes that are essential for *INTS6* mRNA maturation. In the cytosol, parafibromin binds to hSki8 and eEF1Bγ for the destabilization of p53 mRNA, to JAK1/2-STAT1 for STAT1 phosphorylation, and to actinin-2/3 to bundle/cross-link actin filaments. Mice with *CDC73* knockout in the parathyroid develop parathyroid and uterine tumors and are used as a model for HPT-JT syndrome. Conditional deletion of *CDC73* in mesenchymal progenitors results in embryos with agenesis of the heart and liver while its abrogation in mature osteoblasts and osteocytes increases cortical and trabecular bone. Heterozygous germline mutations in *CDC73* are associated with parathyroid carcinogenesis. The rates of *CDC73* mutation and parafibromin loss decrease from parathyroid adenoma to atypical adenoma to carcinoma. In addition, down-regulated parafibromin is closely linked to the tumorigenesis, subsequent progression, or poor prognosis of head and neck, gastric, lung, colorectal, and ovarian cancers, and its overexpression might reverse the aggressiveness of these cancer cells. Therefore, parafibromin might be useful as a biological marker of malignancies and a target for their gene therapy.

## Introduction

Hyperparathyroidism-jaw tumor (HPT-JT) syndrome is an autosomal dominant disease that is characterized by parathyroid tumors, fibro-osseous jaw tumors of the mandible or maxilla, and renal disorders (hamartoma, cystic renal disease, or Wilms’ tumor) and results from a mutation in *CDC73* (also called *HRPT2*) (Carpten et al., 2002). Parafibromin functions as a tumor suppressor in the tumorigenesis and subsequent progression of parathyroid carcinomas. Parafibromin not only forms a PAF1 (polymerase-associated factor 1) complex for transcriptional events and histone modifications during cell growth and survival, but also contributes to cell mobility. In this review, we summarize the gene structure, biological functions, signaling pathways, and phenotypes of *CDC73* knockout mice and the relationship of genetic and expression alterations in *CDC73* with cancer.

### Structure and expression of *CDC73*

*CDC73* maps to human chromosome 1q31.2, spans 18.5 kb, comprises 17 exons, and encodes a 2.7-kb mRNA that is translated into a 60-kDa parafibromin comprising 531 amino acids (Carpten et al., 2002). At the transcriptional level, overexpression of Wilms’ tumor suppressor 1 (WT1) decreases *CDC73* levels and promotes the proliferation of oral squamous cell carcinoma cells by binding to the *CDC73* promoter (Rather et al., 2014). At the translational level, *CDC73* can be targeted and inhibited by miR-182-3p, and its knockdown can reverse the suppressive effects of miR-182-3p inhibitor on the aggressive phenotypes of oral squamous cell carcinoma cells (Guo et al., 2020). Moreover, the oncogenic miR-155 dramatically reduces parafibromin expression in HEK293 cells (Rather et al., 2013). Using northern blot, *CDC73* mRNA expression is detectable in the heart, placenta, brain, lung, skeletal muscle, liver, pancreas, and kidney. In terms of western blot, the 60-kDa form of parafibromin is observed in human adrenal gland, pancreas, heart, and kidney, while the 40-kDa form is found in the skeletal muscle and heart (Carpten et al., 2002). Immunohistochemically, parafibromin positivity has been seen in gastric glandular cells, hepatocytes, glomerular mesangial cell, renal cortex tubules, and hypophysis in both nuclear and nucleocytoplasmic patterns (Porzionato et al., 2006). The expression profile of parafibormin might be closely linked to its biological functions in the cellular compartments.

### Biological functions of parafibromin

**FIGURE 1:**
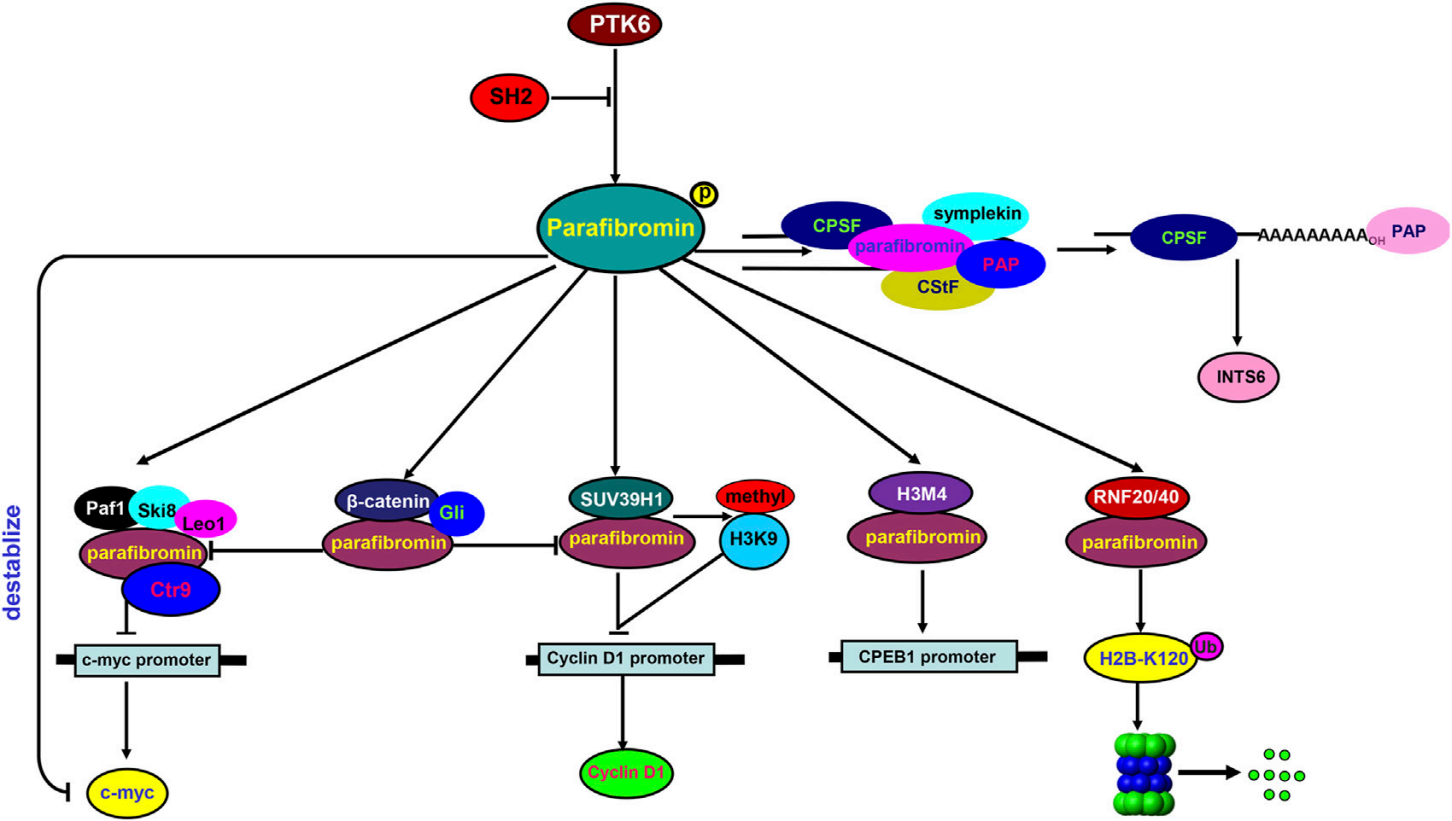
Biological functions of parafibromin in the nucleus In the nucleus, parafibromin interacts with PAF1-Ski8-LEO1-CTR9 to suppress *c-Myc* mRNA expression. It can also bind to a H3M4 and H3K9 methyltransferase complex to downregulate the transcription of *CPEB1* and *cyclin D1*, respectively. The ring finger proteins RNF20/RNF40 bind to parafibromin for monoubiquitination of histone H2B at lysine 120 (H2B-K120). For tyrosine dephosphorylation by SHP2, parafibromin competitively interacts with β-catenin and Gli1 to induce the expression of Wnt target genes, which is attenuated by tyrosine phosphorylation via PTK6 kinase. Parafibromin physically associates with CPSF and CstF complexes, which are required for the maturation of the *INTS6* mRNA 3′ flank.

In the nucleus (Figure 1), parafibromin binds to the human PAF1-LEO1-CTR9 complex and RNA polymerase II for transcription regulation and 3′ flank modification (Rozenblatt-Rosen et al., 2005). Silencing either parafibromin or PAF1 stimulates cell proliferation and enhances *c-myc* expression due to c-myc protein stabilization and *c-myc* promoter activation, with no alleviation of the *c-myc* transcriptional pause (Lin et al., 2008). Parafibromin can also bind to a histone methyltransferase complex for histone H3 methylation at lysine 4 (Lin et al., 2008) and to the promoter of CPEB1 (cytoplasmic polyadenylation element binding protein 1) for the downregulation of CPEB1 expression (Zhang et al., 2010). Parafibromin interacts with the histone methyltransferase SUV39H1 to induce histone H3K9 methylation and suppress cyclin D1 expression (Yang et al., 2010). The heterodimeric parafibromin/RNF20/40 complex acts as an E3 ubiquitin ligase to ubiquitinate histone H2B at lysine 120 (H2B-K120) (Hahn et al., 2012). Upon SHP2-mediated tyrosine dephosphorylation, parafibromin stably interacts with β-catenin to overcome the parafibromin/SUV39H1-induced transrepression and promote the expression of Wnt/β-catenin target genes, such as *c-Myc* and *cyclin D1* (Takahashi et al., 2011). In addition, parafibromin competitively binds to β-catenin and Gli1 and thereby transactivates Wnt- and Hh-target genes; this activity is strengthened by SHP2 phosphatase, but weakened by PTK6 kinase (Kikuchi et al., 2016). As for RNA modification, Parafibromin cleaves the 3′ end of histone mRNA coupled with polyadenylated tails (Farber et al., 2010), and interacts with cleavage stimulation factor (CstF) and the cleavage and polyadenylation specificity factor (CPSF) complex essential for the maturation of the *INTS6* mRNA 3′ flank (Jo et al., 2014). Taken together, parafibromin is involved in the transcriptional regulation of target genes and mRNA maturation via protein–protein complexes.

**FIGURE 2:**
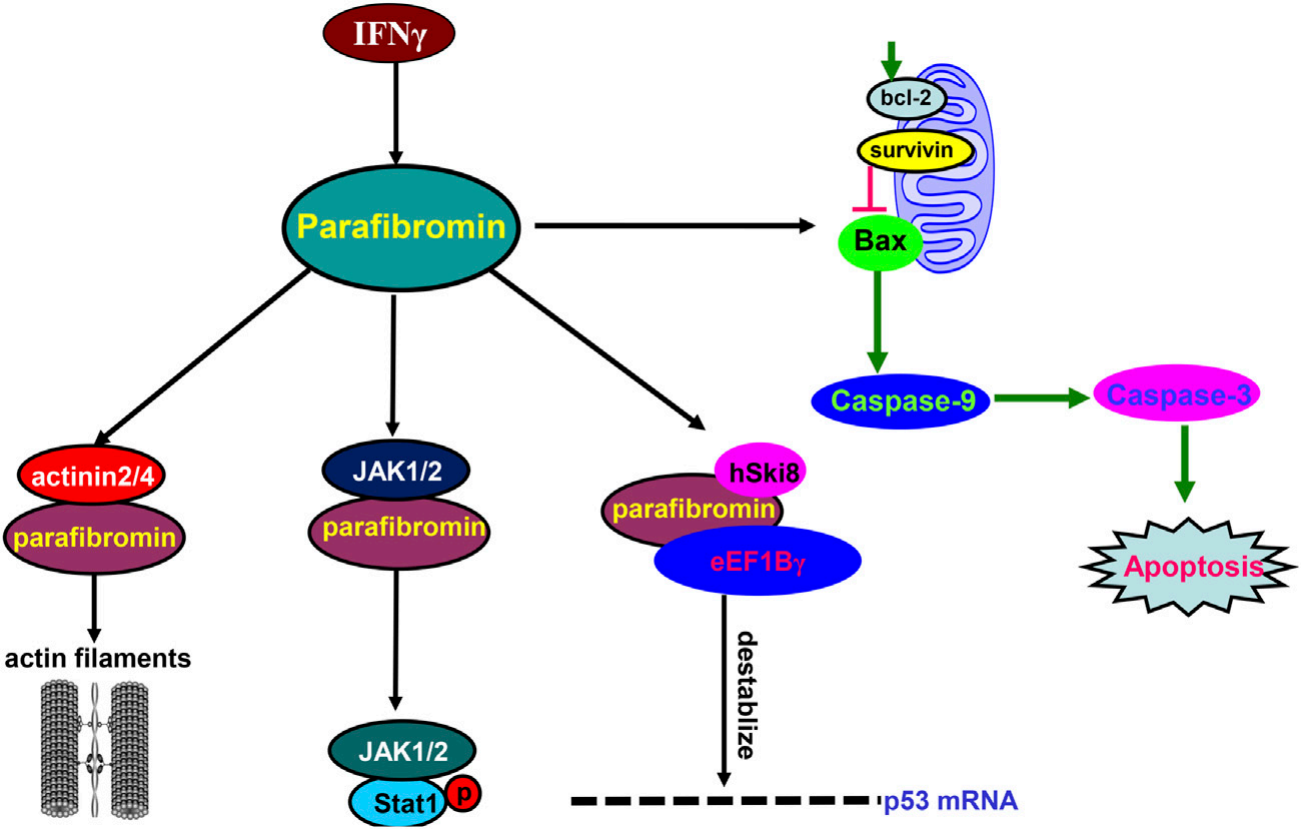
Biological functions of parafibromin in the cytosol. In the cytosol, parafibromin physically binds to eEF1Bγ and hSki8 to destabilize *p53* mRNA. Parafibromin interacts with JAK1/2, promotes the interactions of JAK1-JAK2 and JAK1/2-STAT1, and enhances the tyrosine phosphorylation of STAT1 by JAKs after IFN-γ stimulation. Parafibromin interacts with actinin-2 and actinin-3 to bundle/cross-link actin filaments. Finally, parafibromin causes apoptosis by activating caspase-3 and -9 and downregulating the expression of Bcl-2 and survivin.

In the cytosol (Figure 2), parafibromin physically associates with hSki8 and eEF1Bγ to destabilize *p53* mRNA and inhibit p53-mediated apoptosis (Jo et al., 2014). Parafibromin also interacts with JAK1/2, promotes the formation of the JAK1–JAK2 complex and then the JAK1/2–STAT1 complex, and enhances the JAK-mediated tyrosine phosphorylation of STAT1 upon IFN-γ stimulation (Wei et al., 2015). The N-terminal region of parafibromin binds to actinin-3 and actinin-2 to bundle/cross-link actin filaments for cell mobility in the cytoplasmic compartment (Agarwal et al., 2008), in agreement with the parafibromin expression in the cilia of the bronchial pseudo-stratified columnar epithelium (Xia et al., 2011) and fallopian tube (Shen et al., 2016). Parafibromin overexpression induces apoptosis by activating caspase-3 and -9 and by suppressing survivin and Bcl-2 expression (Zhu et al., 2016). Thus, we suggest that parafibromin contributes to mobility, apoptosis, and proliferation via protein–protein interaction.

### Phenotypes of *CDC73* knockout mice

Reportedly, *CDC73* double knockout was embryonically lethal in mice from E6.5. Temporal deletion of *CDC73* after E8.5 caused growth retardation and extensive apoptosis (Wang et al., 2008). *CDC73* knockout resulted in severe cachexia and death of the adult mice within 20 days. *CDC73* ^−/−^ mouse embryonic fibroblasts (MEFs) underwent apoptosis, whereas *CDC73* ^+/+^ and ^+/−^ MEFs grew normally. The parafibromin/PAF1 complex was found to directly regulate genes related to cell growth and survival, including *H19*, *Hmga1*, *Hmga2*, *Hmgcs2*, *Igf1*, *Igf2*, and *Igfbp4*. The mice with parathyroid-specific deletion of *CDC73* developed parathyroid tumors and could be used as an animal model of HPT-JT syndrome (Walls et al., 2017), and *CDC73* loss in hematopoietic cells was lethal because cell cycle defects in hematopoietic progenitors resulted in bone marrow failure (Saha et al., 2019). Moreover, homozygous knockout of *CDC73* in mesenchymal progenitors (via Dermo1-cre) blocked mesenchymal organ development, such as heart and liver, and displayed extensive apoptosis. The homozygous *CDC73* abrogation in osteoblasts and osteocytes (via Ocn-cre) had no influence on the life span of the mice, but increased cortical and trabecular bone levels and resulted in cytoplasmic RNA accumulation and elevated apoptosis in the osteocytes of the femur (Droscha et al., 2017). These results indicate that *CDC73* knockout mice experience developmental retardation or tumor, possibly via aberrant apoptosis.

### Genetic alterations of *CDC73* during parathyroid carcinogenesis

Biallelic mutation in *CDC73* is strongly related to the malignancy of parathyroid tumors, among which parathyroid cancer (PC) is a rare cancer with an unfavorable prognosis (Hahn et al., 2010). *CDC73* germline mutation causing significant conformational alterations in the conserved C-terminal domain or parafibromin loss (identified as “high-impact mutations”) were substantially more frequently observed in PC patients than in all other individuals with benign tumor. These high-impact mutations were linked to a 6.6-fold higher risk of PC than low-impact mutations. The mutations were mostly nonsense and frameshift, whereas missense mutations were rare and always disrupted the N terminus of parafibromin (Li et al., 2020). *CDC73* mutation rate gradually decreased from PC to atypical adenoma (ATA) to adenoma (AD). A recurrent 2-bp mutation in exon 7 (c.679_680delAG) was responsible for 50% of all identified mutations (Guarnieri et al., 2017). PCs with *CDC73* mutation displayed a high probability of either recurrence or metastasis (Cetani et al., 2013). Wang et al. (2012) identified six mutations in 6 of 13 PC patients, with three being novel and four being germline. PC patients with *CDC73* mutations were more likely to develop recurrence. Copy number alteration in *CDC73* and chromosomal loss at 1p and 13 were only seen in PC, and *CDC73* hypermethylation was not observed in parathyroid tumors (Sulaiman et al., 2012). Both CpG island hyper-methylation and 5′UTR mutation of *CDC73* rarely silenced parafibromin expression in PC as well (Hahn et al., 2010). Additionally, Masi et al. (2014) found that Ile60Asn mutant parafibromin broke its nucleolar localization and was under-expressed due to proteasomal degradation. Overexpression of the Ile60Asn mutant parafibromin failed to suppress c-myc expression, suggesting that Ile60Asn mutant parafibromin lost the ability to down-regulate c-myc expression. In the combination of these findings, *CDC73* mutations are more common during parathyroid carcinogenesis and are closely associated with the recurrence and metastasis of PC by dysfunction of its encoding protein. However, the promoter methylation or mutation of *CDC73* 5′ flank is rarely responsible for its expression loss.

### Parafibromin expression during parathyroid carcinogenesis

Immunohistochemically, parafibromin has been shown to localize to the nucleus and play a tumor-suppressor role. The gradual absence of its nucleolar expression was evident from PC, ATA to AD (Witteveen et al., 2011), consistent with the data of Juhlin et al. (Juhlin et al., 2011). Parafibromin loss in PC was associated it with a 4-fold increased risk of developing local invasion, metastasis and recurrence or metastasis (Witteveen et al., 2011; Kim et al., 2012; Hu et al., 2016). Juhlin et al. (Juhlin et al., 2011) found that the male patients with high-proliferative parathyroid tumors had aberrant parafibromin expression, and PC patients more frequently harbored aberrant parafibromin expression than ATA and AD patients. Parafibromin-negative patients with PC were younger, had larger tumors and more frequent *CDC73* mutation/deletions. PC cells were morphologically characterized by eosinophilic cytoplasm, sheet-like growth, nuclear enlargement, coarse chromatin, perinuclear clear appearance, and branch vasculature (Gill et al., 2019). Although the scoring standard and parafibromin antibodies determined the immunohistochemical and predictive sensitivity and specificity of parafibromin expression (Hu et al., 2019), a meta-analysis showed parafibromin loss was found to be more common in PC patients than in those with parathyroid ATA, AD, and hyperplasia. Parafibromin immunohistochemistry can be useful for the diagnostic and prognostic evaluation of PC in clinicopathological practice (Pyo and Cho, 2019). Zhu et al. (Zhu et al., 2020), who included 193 PC patients from nine studies, demonstrated that parafibromin immunonegativity might be used to reflect the risk of recurrence, metastasis, and death in PC. These findings suggest that parafibromin expression can be employed to indicate the tumorigenesis, progression and prognosis of PC.

### Clinicopathological significance and effects of parafibromin expression on other cancers

Previously, we determined the parafibromin profiles and their clinicopathological significances in head and neck, lung, gastric, colorectal, and ovarian cancers. The downregulated expression of parafibromin in the above-mentioned cancers was negatively correlated with their aggressive variables and adverse prognosis (Zheng et al., 2011; Shen et al., 2016; Zhu et al., 2016; Zheng et al., 2017a; Zheng et al., 2017b). In breast cancer, parafibromin expression was inversely correlated with T stage, clinicopathological stage, local lymphovascular invasion, and C-erbB2 expression (Selvarajan et al., 2008). There was a negative association between the lymph T stage and parafibromin expression in urothelial carcinoma (Karaarslan et al., 2015). Parafibromin expression was positive in 15 cases (50%) of laryngeal squamous cell carcinoma and inversely linked to tumor size and T stage (Cho et al., 2016). Parafibromin expression was immunohistochemically downregulated from normal squamous tissue to dysplasia and then from primary to metastatic cancers of the head and neck and negatively correlated with the N stage, clinicopathological stage, dedifferentiation, and human papillomavirus negativity (Zhang et al., 2015), consistent with the results for colorectal cancer (Zheng et al., 2011). Tongue cancers showed more parafibromin expression than laryngeal cancers (Cho et al., 2016). Parafibromin expression was an independent prognostic factor for the overall or relapse-free survival of head and neck squamous cell carcinomas (Zhang et al., 2015), ovarian cancer (Shen et al., 2016), and colorectal cancer (Zheng et al., 2011). Taken together, downregulated parafibromin expression might be used as a biomarker for tumorigenesis, aggressive behavior, and poor prognosis of non-parathyroid malignancies.

*CDC73* is underexpressed in colorectal cancer and its expression is negatively related to colorectal cancer differentiation at both mRNA and protein levels (Zheng et al., 2017a; Zheng et al., 2017b). Xia et al. (2011) reported that *CDC73* mRNA expression was downregulated in lung cancer in comparison with matched normal tissue, in line with the observation in ovarian cancer (Shen et al., 2016). Bioinformatics analysis showed that *CDC73* mRNA levels were higher in gastric, breast, lung, and ovarian cancers than the corresponding normal tissues and were positively correlated with the differentiation and better prognosis of gastric cancer and with the M stage and clinicopathological stage of lung cancer (55). These findings indicate that the clinicopathological significance of *CDC73* expression depends on cancer type and detection method.

The effects of parafibromin on the aggressive phenotypes of cancer cells determine whether it can be used as a target of gene therapy. In head and neck squamous cell carcinoma cells, parafibromin overexpression suppresses cell proliferation, migration, invasion, and epithelial-mesenchymal transition and induces apoptosis and S arrest (Zheng et al., 2017a). Shen et al. (2016) found that parafibromin overexpression inhibited cell proliferation, anti-apoptosis, migration, and invasion, as well as the chemoresistance to cisplatin in ovarian cancer cells by inhibiting PI3K/Akt and suppressing the expression of VEGF and MMP-9. In colorectal cancer cells, nuclear parafibromin inhibited proliferation and tumor growth and induced apoptosis and cell cycle arrest in colorectal cancer cells, but it was the converse for cytosolic parafibromin. Transcriptomically, nuclear parafibromin inhibited PI3K-Akt and FoxO signaling pathways, while the cytosolic form activated the PI3K-Akt pathway and cell mobility. Thus, the overall results indicate that parafibromin could be used as a gene therapy target in the future (Zheng et al., 2017a).

## Conclusions and future perspectives

In summary, genetic alteration of *CDC73* and altered parafibromin expression both cause HPT-JT, parathyroid, gastric, colorectal, ovarian, lung, and head and neck carcinogenesis. Its down-regulated expression is negatively correlated with the aggressive behaviors and poor prognosis of these cancers. Thus, its overexpression might suppress the proliferation, migration, and invasion of these cancer cells.

According to recent findings concerning *CDC37*, we believe that the genetic study of *CDC73* should be used to protect against and diagnose genetic PC. Aberrant parafibromin expression should be used to predict the tumorigenesis, aggressive behavior, and poor prognosis of malignancies. Wild-type *CDC37* might be used as a molecular target for gene therapy for cancers on the basis of the parafibromin expression status. In the future, the cytosolic function of parafibromin should be thoroughly investigated in cancer cells and in the cilia of the bronchial pseudo-stratified columnar epithelium and fallopian tube.

## Abbreviations

HPT-JT, hyperparathyroidism-jaw tumor; PAF1, polymerase-associated factor 1; WT1, Wilms’ tumor suppressor 1; CPEB1, cytoplasmic polyadenylation element binding protein 1; H2B-K120, histone H2B at lysine 120; CstF, cleavage stimulation factor; CPSF, cleavage and polyadenylation specificity factor; MEFs, mouse embryonic fibroblasts; PC, parathyroid cancer; ATA, atypical adenoma; AD, adenoma.
